# The Mutational Landscape of Early-Onset Breast Cancer: A Next-Generation Sequencing Analysis

**DOI:** 10.3389/fonc.2021.797505

**Published:** 2022-01-21

**Authors:** Angeliki Andrikopoulou, Spyridoula Chatzinikolaou, Ilias Kyriopoulos, Garyfalia Bletsa, Maria Kaparelou, Michalis Liontos, Meletios-Athanasios Dimopoulos, Flora Zagouri

**Affiliations:** ^1^ Department of Clinical Therapeutics, Alexandra Hospital, Medical School, Athens, Greece; ^2^ Hellenic Anticancer Institute, Athens, Greece

**Keywords:** NGS, breast cancer, early-onset, genetic testing, young women

## Abstract

**Background:**

Around 5%–7% of breast cancer cases are diagnosed in women younger than 40, making it the leading cause of female cancer in the 25- to 39-year-old age group. Unfortunately, young age at diagnosis is linked to a more aggressive tumor biology and a worse clinical outcome. The identification of the mutational landscape of breast cancer in this age group could optimize the management.

**Methods:**

We performed NGS analysis in paraffin blocks and blood samples of 32 young patients with breast cancer [<40 years] and 90 older patients during the period 2019 through 2021. All patients were treated in a single institution at the Oncology Department of “Alexandra” Hospital, Medical School, University of Athens, Greece.

**Results:**

Breast tumors were characterized more frequently by HER2 overexpression [25% vs 18.9%], higher ki67 levels [75% vs 61%] and lower differentiation [71.9% vs 60%] in the younger group. PIK3CA [6/20; 30%] and TP53 [6/20; 30%] were the most frequent pathogenic somatic mutations identified in young patients, while one case of BRCA2 somatic mutation [1/20; 5%] and one case of PTEN somatic mutation [1/20; 5%] were also identified. PIK3CA mutations [16/50; 32%] and TP53 mutations [20/50; 40%] were the most common somatic mutations identified in older patients, however other somatic mutations were also reported (ATM, AKT, CHEK2, NRAS, CDKN2A, PTEN, NF1, RB1, FGFR1, ERBB2). As for germline mutations, CHEK2 [3/25; 12%] was the most common pathogenic germline mutation in younger patients followed by BRCA1 [2/25; 8%]. Of note, CHEK2 germline mutations were identified less frequently in older patients [2/61; 3%] among others [BRCA1 (2/61; 3%), ATM (2/61; 3%), APC (1/61; 1,6%) and BRCA2 (1/61; 1,6%)].

**Conclusion:**

We here report the mutational profile identified *via* NGS in patients with early-onset breast cancer compared to their older counterparts. Although the sample size is small and no statistically significant differences were detected, we highlight the need of genetic testing to most patients in this subgroup.

## Introduction

Breast cancer remains the leading cause of cancer among females affecting more than 2 260 000 women worldwide in 2020 (1). Despite the recent advances in diagnosis and treatment, breast cancer remains a major disease burden accounting for 685000 deaths in both sexes and 6.8% of cancer-related mortality (1). Although breast cancer is predominantly a disease of aging, early-onset breast cancer has attracted great interest recently. Young age at diagnosis generally refers to women younger than 40, although the definition may vary between different studies (2–4). Around 5%–7% of breast cancer cases are diagnosed in women younger than 40, making it the leading cause of female cancer in the 25- to 39-year-old age group (2). Even among premenopausal women, the age group <40 years demonstrates an increased risk of breast cancer mortality (4). In the less developed regions like Africa and Middle East, this percentage rises up to 20% due to lack of effective screening. Of note, early childbearing seems to be a risk factor of breast cancer before the age of 35, mainly due to the transient increase in breast cancer risk that occurs around 2 to 7 years following pregnancy (5).

In general, young age at diagnosis is linked to a more aggressive tumor biology and a worse clinical outcome (2–6). Basal-like and HER2-positive tumors are more frequently diagnosed in young women with breast cancer [34.3% and 22% respectively] (2). Azim et al. also observed lower rates of luminal-A tumors [17.2%] compared to over 30% observed in patients aged over 40 (2). Nodal involvement [50%], multifocal disease [27%] and high tumor grade are some of the features more commonly found in younger patients (2, 3). In comparison to the older population, younger patients are diagnosed at a more advanced stage when breast symptoms are profound. These aggressive characteristics lead to an adverse prognosis and a higher mortality rate up to 1.5-fold compared to the older population (4, 7). An analysis of two trial groups, European Organization for Research and Treatment of Cancer (EORTC) and National Surgical Adjuvant Breast and Bowel Project (NSABP), indicated a higher risk of local recurrence in patients younger than 35 years (7).

Recent studies have made an important effort to assess the mutational landscape of breast cancer (8, 9). Specific mutations occur more frequently in this age group. TP53 mutation was the most common somatic mutation [33%] among young patients, followed by PIK3CA [24%] and GATA3 [~25%] somatic mutations. In addition, younger counterparts harbor BRCA1 germline mutations in a greater percentage than the general population [9.4 vs 0.2%] (10). Some studies however failed to confirm the association between the total number of somatic base substitution and the age at diagnosis (8, 9). Accordingly, Azim et al. found no significant differences in the pattern of somatic mutations between pregnant and non-pregnant patients (9). Next-generation sequencing (NGS) has enabled the detection of a broad spectrum of mutations and the characterization of the genomic profile of this population.

The identification of druggable gene mutations in patients with early-onset breast cancer could optimize treatment with novel compounds and enable genetic counselling for the remaining family members. In the era of NGS, multigene panels have become the standard testing option, even though incidental findings and variants with uncertain significance complicate conclusions (11). Apart from cascade testing of patients’ relatives, genetic testing allows the prevention of a second primary malignancy in cancer patients harboring pathogenic germline mutations. We performed NGS analysis in 32 young patients with breast cancer [<40 years] and 90 older patients. The aim of our study is to investigate the frequency and spectrum of pathogenic germline and somatic mutations using NGS in a population of young patients with breast cancer and to compare them with the mutations identified in the older counterparts.

## Materials And Methods

122 women with histologically confirmed breast cancer that underwent NGS in paraffin blocks and blood samples during the period 2019 through 2021 were considered eligible for our study. All women were treated in a single institute at the Oncology Department of “Alexandra” Hospital, Medical School, University of Athens, Greece. An age cut-off of 40 years was used to divide patients into young and old age groups, as previously reported in the literature. Clinicopathological characteristics were retrospectively collected from the patients’ files including age at diagnosis, histological subtype (luminal A, luminal B, HER2-positive, TNBC), histological grade, ki67 expression and disease stage as classified by TNM classification system. Immunohistochemical (IHC) analysis was performed to quantify expression of human epidermal growth factor receptor 2 (HER2), hormone receptors (HR) and Ki67. Estrogen receptor (ER) and progesterone receptor (PR) were considered positive if tumors had more than 1% nuclear-stained cells. HER2 status was considered positive when graded as 3+, while 0 to 1+ were negative and 2+ was an inconclusive result and *in situ* hybridization (ISH) was performed in those cases to confirm positivity. Hormone receptor positive tumors characterized by ki67 expression of over 20% were considered as luminal B. This study was approved by the Institutional Review Board of the Alexandra General Hospital of Athens and performed in accordance with the ethical standards described in the Declaration of Helsinki. An informed consent form was obtained from each of the eligible patients.

### FFPE DNA Sequencing

For breast cancer patients, formalin-fixed paraffin-embedded (FFPE) breast tissues derived from mastectomy, breast-conserving surgery or core biopsy before treatment administration and blood samples were analyzed. Paraffin-embedded breast tissues were cut at slices of 10Mm diameter. FFPE DNA was extracted with the QIAamp DNA FFPE Tissue and libraries were constructed using AmpliSeq for Illumina Comprehensive Panel v3 for the 58 targeted genes that are presented in **Supplementary Table 1**. The genotyping was performed using the Illumina platform (MiSeq, NextSeq500 or NovaSeq) with a median amplicon cover 500x for the 91.39% of the targeted regions. We evaluated predicted pathogenic mutations, based on combined variant characterization by IonReporter (v5.12) (Thermo Scientific) was used. An additional manual inspection was performed using data from OncomineReporter (v4.4) and relevant databases (CinVar, dbSNP, Ensemble, COSMIC, CIVIC, PharmGKB, OMIM, My Cancer Genome, Vasome etc).

### Germline DNA Sequencing

Plasma blood samples were collected in Vacutainer tubes. Within 4 hours after collection, plasma was separated from whole blood samples through centrifugation for 10 min at 3000 rpm at room temperature and stored at −80°C until further use. Genomic DNA was extracted from whole blood using the QIAsymphony DSP DNA Mini Kit and used to prepare indexed libraries to target the sequence of 42 cancer predisposing genes using the Trusight Cancer Panel – Nextera DNA Flex Pre-Enrichment Library Prep (Illumina, San Diego, USA) (**Supplementary Table 2**). Libraries were qualitatively and quantitatively evaluated using a Fragment Analyzer (Advanced Analytical Technologies, Heidelberg, Germany) and sequenced on a MiSeq genetic analyzer (Illumina, Inc., San Diego, CA), according to the manufacturer’s protocols. Annotation was performed against the human reference genome GRCh38 using VariantStudio V.3 (Illumina). The validation of results was performed according to criteria of American College of Medical Genetics – ACMG (12) and NCCN guidelines.

### Statistical Analysis

The statistical analysis was performed with STATA statistical software. Differences between age groups were examined by chi-square test and Fisher’s exact test for categorical variables. The threshold for statistical significance was set at p < 0.05.

## Results

### Clinicopathological Characteristics

Age at diagnosis and histopathological parameters (histological type, grade, ER/PR expression, HER2 expression, ki67, stage) in both age groups are summarized in **Table 1**. Mean age at diagnosis was 35.6 (SD; 4.61) in the young group and 56.94 years (SD; 9.76) in the older group. Invasive ductal carcinoma was the most frequent histologic type of tumor in both groups. Overall, there were no statistically significant differences between the two patient groups. However, tumors were characterized more frequently by HER2 overexpression [25% *vs* 18.9%], higher ki67 levels [75% *vs* 61%] and lower differentiation [71.9% *vs* 60%] in the younger group.

**Table 1:** Clinicopathological characteristics of young (<40) and older (>40) women with breast cancer.

Characteristic	Young women N=32 (N,%)	Older women N=90 (N,%)	Chi-squarep-value
**Mean age at diagnosis, years**	35.6 (4.61)	56.94 (9.76)	
**Stage at diagnosis**			*p = 0.101*
*IA*	5 (15.62%)	22 (24.44%)	
*IB*	0 (0%)	1 (1.11%)	
*IIA*	6 (18.75%)	17 (18.89%)	
*IIB*	5 (15.62%)	10 (11.11%)	
*IIIA*	8 (25%)	10 (11.11%)	
*IIIB*	0 (0%)	6 (6.67%)	
*IIIC*	6 (18.75%)	7 (7.78%)	
*IV*	2 (6.25%)	17 (18.89%)	
**Histology**			*p = 1.000*
*Invasive Ductal Carcinoma*	31 (96.9%)	84 (93.3%)	
*Invasive Lobular Carcinoma*	1 (3.1%)	5 (5.6%)	
*Other*	0 (0.0%)	1 (1.1%)	
**Hormone status**			*p = 0.493*
*Positive*	25 (78.1%)	63 (70%)	
*Negative*	7 (21.9%)	27 (30%)	
			
**ER status**			*p= 0.653*
*Positive*	24 (75%)	62 (68.9%)	
*Negative*	8 (25%)	28 (31.1%)	
**PR status**			*p = 0.516*
*Positive*	23 (71.88%)	57 (63.3%)	
*Negative*	9 (28.12%)	33 (36.7%)	
**HER2 status**			*p = 0.456*
*Positive*	8 (25%)	17 (18.9%)	
*Negative*	24 (75%)	73 (81.1%)	
**Grade**			
*G1*	1 (3.1%)	8 (8.9%)	*p = 0.456*
*G2*	8 (25%)	28 (31.1%)	
*G3*	23 (71.9%)	54 (60%)	
**Ki67**			*p = 0.198*
*<20%*	8 (25%)	35 (38.9%)	
*≥ 20%*	24 (75%)	55 (61.1%)	

**Table 2** summarizes the prevalence of the different molecular subtypes of breast cancer across the two groups. No statistically significant differences were reported between the two populations. Of note, luminal A subtype was more frequently identified in older women [18.75% *vs* 30%] compared to younger patients, while luminal B, HER2-negative subtype was more common in the younger group [43.75% *vs* 28.89%].

**Table 2:** Intrinsic subtypes of breast cancer in younger (<40 years) and older (>40 years) women with breast cancer.

Molecular Subtypes	Young Women N=32 (N,%)	Older Women N=90 (N,%)	
***TNBC***	4 (12.5%)	20 (22.2%)	*P=0.305*
***Luminal A***	6 (18.75%)	27 (30%)	*P=0.254*
***Luminal B, HER2* (–)**	14 (43.75%)	26 (28.89%)	*P=0.132*

### Germline Mutations

Pathogenic germline mutations were identified *via* NGS in 25 young and 61 old patients over 40 years of age. No statistically significant differences were identified. The most common pathogenic germline mutation in younger patients (<40 years) was CHEK2 [3/25; 12%] followed by BRCA1 germline mutation [2/25; 8%]. Other germline mutations identified in younger counterparts were BRCA2 [1/25; 4%] and TP53 mutation [1/25; 4%] (**Table 3**). Of note, c.470T>C was the most common CHEK2 genetic polymorphism detected (rs17879961) in two cases. All germline mutations detected by NGS in the young population are presented in **Table 4**.

**Table 3:** Germline pathogenic mutations of young patients with breast cancer as determined by NGS analysis.

Gene mutation	Genetic polymorphism	NCBI Genomes Browser	Clinical Significance	Frequency
**CHEK2**	c.470T>C p.Ile157Thr	rs17879961	Pathogenic	3
	c.470T>C p.Ile157Thr	rs17879961		
	c.1232G>A p.Trp411*	rs371418985		
**BRCA1**	chr17: c.3157dup p.Glu1053Glyfs*7	rs397509042	Pathogenic	2
	c.3700_3704del p.Val1234Glnfs*8	rs80357609		
**BRCA2**	c.9290_9297del p.Cys3097Phefs*11		Pathogenic	1
**TP53**	c.824G>A p.Cys275Tyr	rs863224451	Pathogenic	1

**Table 4:** Somatic and germline mutations of young women (<40) with breast cancer as determined by NGS.

Patient	Somatic pathogenic mutations	Somatic VUS mutations	Germline Pathogenic mutations	Germline VUS mutations
**1**	–	–	NR	NR
**2**	**PIK3CA** (c.1624G>A p.Glu542Lys rs121913273)	**NF1** (c.7301T>C p.Leu2434Pro Rs1555536128)	NR	NR
**3**	–	–	NR	NR
**4**	–	–	–	–
**5**	–	–	–	**CHEK2** (c.480A>G p.Ile160Met Rs575910805
**6**	**TP53** (c.424_433del p.Pro142Cysfs*25) Chr17: 7578497_7578506del	–	–	–
**7**	**-**	**BRCA2** (c.9613_9614delinsCT p.Ala3205Leu rs276174926)	–	**BRCA2** (c.9613_9614delinsCT p.Ala3205Leu rs276174926 **RAD51D** (c.412A>C p.Asn138His Rs141690729
**8**	NR	NR	–	–
**9**	NR	NR	–	**BRCA2** (c.1342C>T p.Arg448Cys Rs80358422)
**10**	**PIK3CA** (c.3140A>G p.His1047Arg) Rs121913279 **PIK3CA** (c.1090G>A p.Gly364Arg Rs1576935161)	**BRCA1** (c.3743C>T p.A1248V) **TP53** (c.622G>A p.D208N)	–	–
**11**	–	**MYC** (c.1302G>T:p.(Leu434Phe rs145561065) **JAK2** (c.1777-5del) **ERBB4**(c.308G>A p.Arg103His rs754487821)**PIK3CA**(c.3155C>A p.Thr1052Lys)	–	–
**12**	**PIK3CA** (c.3140A>G p.His1047Arg rs121913279)	**RB1** (c.43G>A p.Ala15Thr)	NR	NR
**13**	NR	NR	–	**BRIP1** (c.2285G>A p.Arg762His Rs200960251)
**14**	NR	NR	–	**BRCA1** (c.3649T>C p.Ser1217Pro rs273900712)
**15**	NR	NR	–	**BRCA2** (c.8386C>T p.Pro2796Ser Rs146120136)
**16**	**PIK3CA** (c.3140A>G p.His1047Arg rs121913279) **BRCA2** (c.9290_9297del p.Cys3097Phefs*11)	**AR** (c.1520G>A p.Gly507Asp Rs768030110) **JAK2** (c.779A>T p.Asn260Ile rs774355597)	**BRCA2** (c.9290_9297del p.Cys3097Phefs*11)	–
**17**	**TP53** (chr17: c.488A>G p.Tyr163Cys rs148924904)	**RAD50** (chr5: c.1094G>A p.Arg365Gln rs146370443) **TP53** (chr17: c.562_564delinsAA p.Leu188Lysfs*59) **CCND1** (chr11: c.724-2A>C)	–	–
**18**	**PIK3CA** (chr3: c.3140A>T p.His1047Leu Rs121913279)	**TP53** (chr17: c.815T>C p.Val272Ala rs28934573) **IDH1** (chr2: c.949C>T p.R317C rs754290687) **CTNNB1** (chr3: c.1206del p.Thr404Leufs*11) **KMT2C** (chr7: c.3521A>G p.Gln1174Arg rs771444390)	NR	NR
**19**	**TP53** (chr17: c.586C>T p.Arg196* rs397516435)	–	NR	NR
**20**	**TP53** (chr17: c.743G>A p.Arg248Gln rs11540652)	**KMT2C** *(chr7:*c.4873G>A p.Glu1625Lys)	–	–
**21**	**TP53** (exon8: c.824G>A p.Cys275Tyr)	**ROS1** (exon5: c.433A>C p.Thr145Pro) **RET** (exon9: c.1684A>T p.Thr562Ser)	**TP53** (c.824G>A p.Cys275Tyr rs863224451)	–
**22**	**TP53** (chr17: c.990del p.Gln331Argfs*14)	**MET** (c.4090C>T p.Pro1364Ser rs765332671) **NF1** (c.563C>A p.Ala188Glu)	–	–
**23**	NR	NR	–	–
**24**	NR	NR	–	–
**25**	NR	NR	–	**ATM** (c.7475T>G p.Leu2492Arg rs56399857)
**26**	**PTEN (**exon2: c.99_100ins p.Ala34fs)	**FGFR3** (exon17: c.2272G>A p.Asp758Asn) **ROS1** (exon10: c.1135C>G p.Gln379Glu) **BRAF** (exon9: c.1159G>A p.Gly387Arg) **TP53** (exon7: c.691del p.Thr231ProfsTer16)	NR	NR
**27**	NR	NR	**CHEK2** (c.1232G>A p.Trp411* rs371418985)	–
**28**	NR	NR	**CHEK2** (c.470T>C p.Ile157Thr rs17879961)	–
**29**	NR	NR	**BRCA1** (c.3700_3704del p.Val1234Glnfs*8 rs80357609) **CHEK2** (c.470T>C p.Ile157Thr rs17879961)	**BRCA2** (c.352C>T pArg118Cys rs375125172)
**30**	**PIK3CA** (c.1637A>G p.Gln546Arg)	NR	–	**CHEK2** (c.1175C>T p.Ala392Val rs373073383)
**31**	NR	NR	**BRCA1** (chr17: c.3157dup p.Glu1053Glyfs*7 Rs397509042)	–
**32**	–	–	NR	NR

NR, Not Reported.

Pathogenic germline mutations identified in older patients are presented in **Table 5**. The most frequent germline mutations detected were BRCA1 [2/61; 3%], ATM [2/61; 3%] and CHEK2 [2/61; 3%] mutations. Other germline mutations detected less frequently were APC [1/61; 1,6%] and BRCA2 [1/61; 1,6%] mutations. All pathogenic and non-pathogenic germline mutations identified in the older (>40 years) population are presented in **Table 6**.

**Table 5:** Germline pathogenic mutations of old patients (>40 years) with breast cancer as determined by NGS analysis.

Gene mutation	Genetic polymorphism	NCBI Genomes Browser	Clinical Significance	Frequency
**BRCA1**	chr17: c.5467G>A p.Ala1823Thr	rs80357212	Pathogenic	2
	chr17: c.3157dup p.Glu1053Glyfs*7	rs397509042		
**CHEK2**	chr22: c.470T>C p.Ile157Thr	rs17879961	Pathogenic	2
	chr22: c.499G>A p.Gly167Arg	rs72552322		
**ATM**	chr11: c.368del p.Tyr123Leufs*6	rs730881296	Pathogenic	2
	chr11: c.368del p.Tyr123Leufs*6	rs730881296		
**BRCA2**	chr13: c.5110_5113del p.Arg1704* rs879254123	rs879254123	Pathogenic	1
**APC**	chr5: c.3920T>A p.Ile1307Lys	rs1801155	Pathogenic	1

**Table 6:** Somatic and germline mutations of old women (>40) with breast cancer as determined by NGS.

Patient	Somatic pathogenic mutations	Somatic VUS mutations	Germline Pathogenic mutations	Germline VUS mutations
**1**	–	**FGFR1** (chr8: c.2192_*del p.Lys731_Arg732fs*159)	–	**CHEK2** (chr22: c.190G>A p.Glu64Lys rs141568342)
**2**	NR	NR	**CHEK2** (chr22: c.470T>C p.Ile157Thr rs17879961)	–
**3**	NR	NR	**CHEK2** (chr22: c.499G>A p.Gly167Arg rs72552322)	–
**4**	**ATM** (chr11: c.494T>G p.Leu165*)	**ATM** (chr11: c.482A>C p.Gln161Pro rs587780625)	–	–
**5**	**AKT1** (chr14 c.49G>A p.Glu17Lys rs121434592)	**PTEN** (chr10: c.481A>G p.Arg161Gly) **RAD50** (chr5: c.443A>G p.Lys148Arg)	–	–
**6**	NR	NR	–	–
**7**	NR	NR	–	–
**8**	**PIK3CA** (chr3: c.1633G>A p.Glu545Lys rs104886003)	**BRCA2** (chr13: c.3073A>G p.Glu1025Lys rs80358550) **PDGFRA** (chr4: c.632C>T p.Thr211Ile) **KMT2C** (chr7: c.5976A>T p.Glu1992Asp) **MTOR** (chr1: c.5554G>A p.Glu1852Lys rs745409542)	NR	NR
**9**	NR	NR	**BRCA1** (chr17: c.5467G>A p.Ala1823Thr rs80357212)	–
**10**	–	–	–	**PMS2** (chr7: c.1999G>A p.Glu667Lys Rs587780045)
**11**	NR	NR	–	–
**12**	NR	NR	**ATM** (chr11: c.368del p.Tyr123Leufs*6 rs730881296)	**RAD51D** (chr17: c.270T>A p.Asp90Glu rs1567728766)
**13**	NR	NR	–	**RAD51C** (chr17: c.80T>C p.Leu27Pro Rs587781309)
**14**	NR	NR	–	**BRCA1** (chr17: c.1881C>G p.Val627= rs80356838)
**15**	**CDKN2A** (chr9: c.219_235del p.Gly74Serfs*81)	**TP53** (chr17: c.815T>C p.Val272Ala rs28934573)	–	–
**16**	–	**PIK3CA** (chr3: c.312_323del p.Val105_Arg108del) **FGFR3** (chr4: c.1936G>A p.Asp646Asn rs56266857)	–	–
**17**	NR	NR	–	–
**18**	–	**ATM** (chr11: c.2057T>A p.Leu686His rs1239416977)	–	**ATM** (chr11: c.2057T>A p.Leu686His rs1239416977)
**19**	**PIK3CA** (chr3: c.1624G>A p.Glu542Lys rs121913273) **CHEK2** (chr22: c.470T>C p.Ile157Thr rs17879961)	**NOTCH1** (chr9: c.6184G>A p.Glu2062Lys)	–	–
**20**	NR	NR	–	–
**21**	NR	NR	–	–
**22**	NR	NR	–	–
**23**	NR	NR	–	–
**24**	NR	NR	–	–
**25**	NR	NR	–	**ATM** (chr11: c.5329G>A p.Val1777Ile rs1064794192)
**26**	NR	NR	**BRCA2** (chr13: c.5110_5113del p.Arg1704* rs879254123)	–
**27**	NR	NR	–	–
**28**	NR	NR	–	–
**29**	NR	NR	–	–
**30**	**PIK3CA** (exon10: c.1633G>A p.Glu545Lys)	**BRCA2** (exon22: c.8808G>C p.Leu2936Phe) **ERBB4** (exon7: c.751A>T p.Asn251Tyr) **KIT** (exon11: c.1688T>A p.Ile563Lys)	NR	NR
**31**	NR	NR	–	–
**32**	NR	NR	–	–
**33**	NR	NR	–	–
**34**	NR	NR	–	–
**35**	–	**ATM** (exon56: c.8187A>C p.Gln2729His rs587781946) **ROS1** (exon5: c.433A>C p.Thr145Pro) **JUN** (exon1: c.872A>G p.Asn291Ser)	**APC** (chr5: c.3920T>A p.Ile1307Lys Rs1801155)	**ATM** (chr11: c.8187A>C p.Gln2729His rs587781946)
**36**	–	**ROS1** (exon5: c.433A>C p.Thr145Pro) **NOTCH1** (exon34: c.7487_7489del p.Asn2496del)	NR	NR
**37**	NR	NR	–	–
**38**	NR	NR	–	–
**39**	NR	NR	–	–
**40**	–	**BRCA2** (exon26: c.9578C>T p.Thr3193Ile) **ATM** (exon41: c.6067G>A p.Gly2023Arg) **ESR1** (exon5: c.975del: p.Ile326fs)	NR	NR
**41**	NR	NR	–	–
**42**	**TP53** (chr17: c.824G>A p.Cys275Tyr rs863224451)	**KMT2C** (chr7: c.7826G>A p.Arg2609Gln rs746659124) **RB1** (chr13: c.1988A>G p.Asn663Ser rs1007286459) **NOTCH1** (chr9: c.2453T>C p.Leu818Pro)	–	–
**43**	**TP53** (chr17: c.536A>G p.His179Arg rs1057519991)	**ROS1** (chr6: c.6584C>G p.Thr2195Ser rs140284927) **TP53** (chr17: c.847C>T p.Arg283Cys rs149633775)	–	**TP53** (chr17: c.847C>T p.Arg283Cys rs149633775) **PMS2** (chr7: c.566A>C p.His189Pro rs876660330)
**44**	**PIK3CA** (chr3: c.1633G>A p.Glu545Lys rs104886003) **TP53** (chr17: c.797G>A p.Gly266Glu rs193920774) **PTEN** (chr10: c.853dup p.Glu285Glyfs*13)	**MSH2** (chr2: n.1717G>A p.Glu561Lys rs63750328) **NOTCH1** (chr9: c.1816G>A p.Glu606Lys)	NR	NR
**45**	**PIK3CA** (chr3: c.1633G>A p.Glu545Lys rs104886003) **TP53** (chr17: c.722C>T p.Ser241Phe rs28934573) **NRAS** (chr1: c.178G>A p.Gly60Arg rs1557982817)	**BRCA2** (c.3478A>G p.Arg1160Gly) **BRCA2** (c.3476G>A p.Cys1159Tyr) **CDKN2A** (c.28C>T p.Arg10Trp) **RET** (c.2302G>A p.Glu768Lys) **NF1** (c.7454C>G p.Ala2485Gly) **STK11** (c.1222G>A p.Gly408Ser rs749463771) **STK11** (c.196G>A p.Val66Met)	–	–
**46**	**TP53** (exon8: c.853G>A p.Glu285Lys)	**NF1** (exon4: c.304A>G p.Met102Val) **ROS1** (exon5: c.433A>C p.Thr145Pro)	NR	NR
**47**	**NF1** (exon43: c.6590_6591del p.Phe2197fs) **RB1** (exon11: c.1064_1065del p.Arg355fs)	**TP53** (exon7: c.683_686del p.Asp228fs)	NR	NR
**48**	**TP53** (exon7: c.742C>T p.Arg248Trp)	**AR** (exon1: c.1481A>C p.Glu494Ala) **ERBB2** (exon12: c.1460G>A p.Arg487Gln) **NF1** (exon50: c.7395T>A p.Asp2465Glu) **ROS1** (exon6: c.500G>A p.Arg167Gln)	–	–
**49**	–	**CHEK2** (exon2: c.32A>C p.Gln11Pro) **RAD50** (exon7: c.980G>A p.Arg327His)	NR	NR
**50**	**PIK3CA** (exon10: c.1636C>A p.Gln546Lys)	**ROS1** (exon6: c.500G>A p.Arg167Gln) **NF1** (exon58: c.8383G>A p.Asp2795Asn)	NR	NR
**51**	NR	NR	–	–
**52**	**TP53** (exon8: c.817C>T p.Arg273Cys)	**ROS1** (exon36: c.5824C>T p.Arg1942Trp) **KMT2C** (exon52: c.13231G>C p.Gly4411Arg)	NR	NR
**53**	NR	NR	–	–
**54**	NR	NR	–	**BRCA2** (chr13: c.710A>G p.Asp237Gly Rs730881506)
**55**	**PIK3CA** (exon21: c.3140A>G p.His1047Arg) **TP53** (exon7: c.743G>A p.Arg248Gln)	**ROS1** (exon6: c.500G>A p.Arg167Gln)	–	–
**56**	**PIK3CA** (exon10: c.1624G>A p.Glu542Lys)	**RAD50** (exon16: c.2604T>G p.Asn868Lys)	NR	NR
**57**	**TP53** (exon7: c.714_715insT p.Asn239Ter)	**ROS1** (exon42: c.6733G>A p.Gly2245Ser) **ROS1** (exon5: c.433A>C p.Thr145Pro)	NR	NR
**58**	**TP53** (exon7: c.743G>A p.Arg248Gln)	**ROS1** (exon6: c.500G>A p.Arg167Gln) **KMT2C** (exon25: c.3875G>A p.Arg1292Gln)	–	–
**59**	**TP53** (exon3: c.85_86del p.Asn29GlnfsTer13)	**BRCA2** (exon27: c.9867T>G p.Phe3289Leu) **ROS1** (exon5: c.433A>C p.Thr145Pro)	–	**BRCA2** (chr13: c.9867T>G p.Phe3289Leu)
**60**	**PIK3CA** (chr3: c.1357G>A p.Glu453Lys rs1057519925)	–	NR	NR
**61**	NR	NR	–	–
**62**	NR	NR	–	**MSH6** (chr2: c.3104G>A p.Arg1035Gln rs730881801)
**63**	–	**RAD50** (chr5: c.3929A>G p.Asn1310Ser rs753468016)	NR	NR
**64**	–	**PIK3CA** (chr3: c.1259_1264del p.Cys420_Pro421del)	NR	NR
**65**	NR	NR	–	–
**66**	–	**MYC** (exon2: c.77A>G p.Asn26Ser) **NOTCH1** (exon23: c.3741G>C p.Gln1247His)	–	–
**67**	NR	NR	**BRCA1** (chr17: c.3157dup p.Glu1053Glyfs*7 Rs397509042)	–
**68**	**PIK3CA** (exon21: c.3140A>G p.His1047Arg)	–	NR	NR
**69**	NR	NR	–	–
**70**	–	**ALK** (exon9: c.1787T>C p.Met596Thr)	NR	NR
**71**	**PIK3CA** (exon21: c.3140A>G p.His1047Arg) **ERBB2** (exon19: c.2264T>C p.Leu755Ser)	**RAD50** (exon7: c.980G>A p.Arg327His) **TP53** (exon4: c.304A>T p.Thr102Ser) **NF1** (exon21: c.2573C>G p.Ser858Cys)	NR	NR
**72**	**PIK3CA** (chr3: c.1633G>A p.Glu545Lys rs104886003) **TP53** (chr17: c.559+1G>A rs1131691042)	–	NR	NR
**73**	**TP53** (exon6: c.638G>T p.Arg213Leu)	**KDR** (exon13: c.1875G>C p.Leu625Phe) **CDK4** (exon7: c.793G>A p.Glu265Lys) **CDK4** (exon7: c.792G>A p.Met264Ile)	NR	NR
**74**	**TP53** (exon5: c.455C>T p.Pro152Leu)	**ROS1** (exon5: c.433A>C p.Thr145Pro) **MET** (exon21: c.4144C>T p.Pro1382Ser) **KMT2C** (exon38: c.8075A>C p.Asp2692Ala) **MYC** (exon2: c.77A>G p.Asn26Ser)	–	–
**75**	**PIK3CA** (exon2: c.115G>A p.Glu39Lys) **TP53** (exon8: c.818G>T p.Arg273Leu)	–	NR	NR
**76**	**TP53** (exon7: c.681_682insT p.Asp228Ter)	**MTOR** (exon40: c.5687G>A p.Arg1896Gln) **ROS1** (exon42: c.6733G>A p.Gly2245Ser) **KMT2C** (exon10: c.1315A>G p.Ile439Val) **IDH2** (exon1: c.68C>A p.Pro23Gln)	NR	NR
**77**	**FGFR1** (exon13: c.1731C>G p.Asn577Lys)	**KDR** (exon19: c.2695G>T p.Val899Phe) **RAD50** (exon7: c.980G>A p.Arg327His) **ROS1** (exon5: c.433A>C p.Thr145Pro)	NR	NR
**78**	NR	NR	–	–
**79**	NR	NR	–	–
**80**	**TP53** (exon6: c.626_627del p.Arg209LysfsTer6)	**PIK3CA** (exon8: c.1316G>C p.Gly439Ala) **CDK6** (exon3: c.269G>C p.Arg90Thr) **JAK2** (exon23: c.3070G>A p.Glu1024Lys)	–	–
**81**	**PIK3CA** (exon10 c.1633G>A p.Glu545Lys)	**PALB2** (exon4: c.1544A>G p.Lys515Arg) **CHEK2** (exon15: c.1556G>T p.Arg519Leu) **EGFR** (exon3: c.368C>T p.Ser123Phe)	NR	NR
**82**	NR	NR	**ATM** (chr11: c.368del p.Tyr123Leufs*6 rs730881296)	–
**83**	NR	NR	–	–
**84**	**CHEK2** (exon4: c.470T>C p.Ile157Thr)	**CCNE1** (exon9: c.779A>T p.Asn260Ile) **IDH2** (exon11: c.1321A>G p.ile441Val)	NR	NR
**85**	–	**CCNE1** (exon9: c.779A>T p.Asn260Ile)	NR	NR
**86**	**PIK3CA** (exon21: c.3140A>G p.His1047Arg) **TP53** (exon7: c.742C>T p.Arg248Trp)	**ERBB4** (exon9: c.1088A>T p.Asn363Ile) **CCND1** (exon1: c.189G>C p.Trp63Cys)	NR	NR
**87**	–	–	–	–
**88**	**AKT1** (chr14: c.49G>A p.Glu17Lys Rs121434592) **TP53** (chr17: c.824G>A p.Cys275Tyr rs863224451)	–	–	–
**89**	**TP53** (chr17: c.614A>G p.Tyr205Cys rs1057520007)	–	NR	NR
**90**	**PIK3CA** (chr3: c.3140A>G p.His1047Arg rs121913279)	**TP53** (chr17: c.377A>G p.Tyr126Cys rs587780625) **CDKN2A** (chr9: c.323C>T p.Ala108Val) **RAD50** (chr5: c.3778C>T p.Arg1260Cys rs1381323366) **MET** (chr7: c.2996A>C p.Glu999Ala)	NR	NR

NR, Not Reported.

### Somatic Mutations

Pathogenic somatic mutations were identified *via* NGS in 20 young and 50 old patients. PIK3CA [6/20; 30%] and TP53 [6/20; 30%] were the most frequent pathogenic somatic mutations identified in young patients. Of note, the genetic variant c.3140A>G was the most common PIK3CA mutation identified (rs121913279). There was one case of BRCA2 somatic mutation [1/20; 5%] and one case of PTEN somatic mutation [1/20; 5%]. All pathogenic somatic mutations detected in the young subgroup are presented in **Table 7**. All somatic mutations both pathogenic and variants of unknown significance identified in young (<40 years) patients with breast cancer are presented in **Table 4**.

**Table 7:** Somatic pathogenic mutations of young patients with breast cancer as determined by NGS analysis.

Gene mutation	Genetic polymorphism	NCBI genomes browser	Clinical significance	Frequency
**PIK3CA**	c.1624G>A p.Glu542Lys	rs121913273	Pathogenic	6
	c.3140A>G p.His1047Arg	rs121913279		
	c.1090G>A p.Gly364Arg	rs1576935161		
	c.3140A>G p.His1047Arg rs121913279	rs121913279		
	c.3140A>G p.His1047Arg rs121913279)	rs121913279		
	c.3140A>T p.His1047Leu	rs121913279		
	c.1637A>G p.Gln546Arg	–		
**TP53**	c.424_433del p.Pro142Cysfs*25	–	Pathogenic	6
	c.488A>G p.Tyr163Cys	rs148924904		
	c.586C>T p.Arg196*	rs397516435		
	c.743G>A p.Arg248Gln	rs11540652		
	c.824G>A p.Cys275Tyr	rs863224451		
	c.990del p.Gln331Argfs*14	–		
**BRCA2**	c.9290_9297del p.Cys3097Phefs*11	–	Pathogenic	1
**PTEN**	c.99_100ins p.Ala34fs	–	Pathogenic	1

Pathogenic somatic mutations identified in older patients are presented in **Table 8**. No statistically significant differences were detected between the somatic mutations identified in young versus older patients. The most frequent somatic mutations include PIK3CA mutations [16/50; 32%] and TP53 mutations [20/50; 40%]. Other mutations detected by NGS include: ATM [1/50; 2%], AKT [2/50; 4%], CHEK2 [2/50; 4%], NRAS [1/50; 2%], CDKN2A [1/50; 2%], PTEN [1/50; 2%], NF1 [1/50; 2%], RB1 [1/50; 2%], FGFR1 [1/50; 2%] and ERBB2 [1/50; 2%]. The most common PIK3CA mutation in older patients was c.1633G>A p.Glu545Lys in chromosome 13 (rs104886003) [6/16], followed by c.3140A>G p.His1047Arg (rs121913279) [5/16] and c.1624G>A p.Glu542Lys (rs121913273) [2/16]. All pathogenic somatic mutations and somatic mutations of unknown significance identified in the older (>40 years) population are presented in **Table 6**.

**Table 8:** Somatic pathogenic mutations of old patients (>40 years) with breast cancer as determined by NGS analysis.

Gene mutation	Genetic polymorphism	NCBI Genomes Browser	Clinical Significance	Frequency
**PIK3CA**	chr3: c.1633G>A p.Glu545Lys	rs104886003	Pathogenic	16
	chr3: c.1624G>A p.Glu542Lys	rs121913273		
	chr3: c.1633G>A p.Glu545Lys	rs104886003		
	chr3: c.1633G>A p.Glu545Lys	rs104886003		
	chr3: c.1633G>A p.Glu545Lys	rs104886003		
	exon10: c.1636C>A p.Gln546Lys			
	chr3: c.3140A>G p.His1047Arg	rs121913279		
	chr3: c.1624G>A p.Glu542Lys	rs121913273		
	chr3: c.1357G>A p.Glu453Lys	rs1057519925		
	chr3: c.3140A>G p.His1047Arg	rs121913279		
	chr3: c.3140A>G p.His1047Arg	rs121913279		
	chr3: c.1633G>A p.Glu545Lys	rs104886003		
	exon2: c.115G>A p.Glu39Lys			
	chr3: c.1633G>A p.Glu545Lys)	rs104886003		
	chr3: c.3140A>G p.His1047Arg)	rs121913279		
	chr3: c.3140A>G p.His1047Arg	rs121913279		
**ATM**	chr11: c.494T>G p.Leu165*		Pathogenic	1
**AKT1**	chr14 c.49G>A p.Glu17Lys	rs121434592	Pathogenic	2
	chr14: c.49G>A p.Glu17Lys	Rs121434592		
**CDKN2A**	chr9: c.219_235del p.Gly74Serfs*81		Pathogenic	1
**CHEK2**	chr22: c.470T>C p.Ile157Thr	rs17879961	Pathogenic	2
	exon4: c.470T>C p.Ile157Thr)			
**TP53**	chr17: c.824G>A p.Cys275Tyr	rs863224451	Pathogenic	20
	chr17: c.536A>G p.His179Arg	rs1057519991		
	chr17: c.797G>A p.Gly266Glu	rs193920774		
	chr17: c.722C>T p.Ser241Phe	rs28934573		
	exon8: c.853G>A p.Glu285Lys			
	exon7: c.742C>T p.Arg248Trp)			
	exon8: c.817C>T p.Arg273Cys			
	exon7: c.743G>A p.Arg248Gln			
	exon7: c.714_715insT p.Asn239Ter			
	exon7: c.743G>A p.Arg248Gln			
	exon3: c.85_86del p.Asn29GlnfsTer13			
	exon6: c.638G>T p.Arg213Leu			
	chr17: c.559+1G>A	rs1131691042		
	exon5: c.455C>T p.Pro152Leu			
	exon8: c.818G>T p.Arg273Leu			
	exon7: c.681_682insT p.Asp228Ter			
	exon6: c.626_627del p.Arg209LysfsTer6			
	exon7: c.742C>T p.Arg248Trp			
	chr17: c.614A>G p.Tyr205Cys	rs1057520007		
	chr17: c.824G>A p.Cys275Tyr	rs863224451		
**PTEN**	chr10: c.853dup p.Glu285Glyfs*13			1
**NRAS**	chr1: c.178G>A p.Gly60Arg	rs1557982817		1
**NF1**	exon43: c.6590_6591del p.Phe2197fs			1
** RB1**	exon11: c.1064_1065del p.Arg355fs			1
**ERBB2**	exon19: c.2264T>C p.Leu755Ser			1
**FGFR1**	exon13: c.1731C>G p.Asn577Lys)			1

## Discussion

We here provide the mutational profile of younger patients with breast cancer below 40 years of age versus their older counterparts as determined *via* powerful next-generation sequencing (NGS) analysis. No statistically significant differences were observed between the histopathological characteristics and the intrinsic molecular subtypes of breast cancer between the two groups although the sample size of the entire cohort is relatively small and the data is limited. However, younger patients tended to present with HER2-overexpressing [25% vs 18.9%], highly proliferating [ki67 ≥ 20%: 75% vs 61%] and high grade [71.9% vs 60%] tumors in a higher percentage. In contrast, luminal A breast cancer was more frequently identified in older women [18.75% vs 30%] compared to younger patients. PIK3CA and TP53 were the most frequent pathogenic somatic mutations identified in both subgroups harboring around 30% of the cases analyzed. These results are in agreement with previous studies that report an incidence of PIK3CA and TP53 somatic mutations in ~25-30% of breast tumors (8, 13, 14). All of the other somatic mutations detected (ATM, AKT, CHEK2, NRAS, CDKN2A, PTEN, NF1, RB1, FGFR1, ERBB2, PTEN, BRCA2) were identified in a percentage <2% of breast tumors as previously reported in the literature (8, 14). Indeed, NGS studies have revealed that the majority of cancer genes are mutated at frequencies of less than 5 or even 2% revealing a “long tail” of rare yet recurrent breast cancer genes (14). The most common PIK3CA somatic mutation identified was c.3140A>G (rs121913279) in younger women, whereas c.1633G>A (rs104886003) was the one most frequently detected in older patients. The most common somatic PIK3CA mutations are c.1624G>A, c.1633G>A, c.3140A>G and c.3140A>T, accounting for more than 90% of all mutations (15).

As for germline mutations, the high prevalence of CHEK2 [3/25; 12%] and BRCA1/2 germline mutations [3/25; 12%] in younger patients (<40 years) is of clinical significance. It has been shown that BRCA1 mutation and CHEK2 mutations, especially the CHEK21100delC germline mutation occurs more frequently in younger patients. Approximately 2% of patients with breast cancer carry a BRCA1 mutation, but among young patients this percentage rises up to 6-7% (16, 17). In addition, BRCA1 mutation carriers tend to be diagnosed at younger ages than noncarriers (16, 17). It has been shown that CHEK2 germline mutation carriers are at increased risk of developing female breast cancer with a predisposition to ER-positive disease and colon cancer (18). CHEK2 is a tumor suppressor gene that encodes a serine/threonine kinase, the CHK2, that is involved in multiple cellular pathways such as DNA repair, cell cycle and apoptosis. Various aberrations in CHEK2 gene have been recorded, including 1100delC, I157T, R117G, I160M, G167R and G167A (19). The most common germline mutation identified in our study was c.470T>C (p.Ile157Thr). It has been shown that the I157T variant is associated with breast cancer [odds ratio (OR) 1.4; *p*=0.02], prostate (OR 1.7; *p*=0.002), kidney (OR 2.1; *p*=0.0006), colon (OR 2.0; *p*=0.001) and thyroid (OR 1.9; *p*=0.04) cancer (19). A two-fold risk for breast cancer in carriers of CHEK2 mutations compared with noncarriers has been reported with a lifetime risk of breast cancer of approximately 15% to 20% (20). The risk of developing breast cancer in CHEK2 mutation carriers is associated with family history and increases when the carriers have first- and second-degree relatives who are affected (21). Of note, the rate of CHEK2 germline mutations was higher in the younger population (<40) compared to the older subgroup [12% vs 3%]. Whether CHEK2 mutations could be associated with early-onset breast cancer should be further investigated.

A single case of TP53 germline mutation was detected in a patient that belonged to the young subgroup. Heterozygous germline TP53 variants are the genetic cause of Li-Fraumeni syndrome (LFS), a hereditary cancer predisposition syndrome associated with very early-onset female breast cancer, commonly occurring before 31 years. Our patient that carried the c.824G>A (rs863224451) TP53 germline mutation was diagnosed with locally advanced, non-luminal HER2-positive breast cancer at the age of 32 and had a positive family history with a mother diagnosed with uterine sarcoma and a father diagnosed with hepatocellular carcinoma. The c.824G>A pathogenic mutation located in coding exon 7 of the TP53 gene has been identified as a germline alteration in patients with LFS and has therefore been classified as pathogenic (22). In germline TP53 pathogenic variant carriers, breast cancer risk increases significantly after the second decade, rises up to 20%–30% under the age 31, reaches a peak between 25–35 years and drops after 40 years of age, while the cumulative risk reaches a plateau before 60 (23). Women who carry germline mutations in the *TP53* gene have a cumulative risk of developing breast cancer of up to 85% by the age of 60, whereas approximately 5–8% of women presenting with breast cancer under 30 years old have a germline TP53 gene mutation. It has been shown that TP53 germline mutations are associated with tumors characterized by high grade, HER2 overexpression in 60%–83% of the cases and multifocality (23). Guidelines for surveillance of TP53 pathogenic variant carriers have incorporated the annual breast MRI from the age of 20 onwards, although the option of risk-reducing mastectomy may be discussed in certain cases. Overall, TP53 germline testing should systematically be applied on all patients diagnosed with invasive breast cancer or ductal carcinoma *in situ* (DCIS) before 31 years of age.

Two germline mutations were detected only in the older breast cancer patients but not in their younger counterparts: ATM [2/61; 3%] and APC [1/61; 1,6%] pathogenic mutations. In agreement with these results, there is evidence that ATM germline mutations are not associated with familial breast cancer or diagnosis at a younger age (24). *ATM* is the fifth DNA repair gene, together with *BRCA1*, *BRCA2*, *TP53* and *CHEK2* shown to be involved in breast cancer predisposition (25). The overall relative risk of breast cancer in carriers is estimated to be 2.23-3.9 according to previous studies and is higher (~4.9) in women under 50 years of age (25). Although the family history of breast cancer is slightly higher in ATM mutation carriers, there was no difference in the median age at diagnosis between ATM carriers (48.6 years) and patients without ATM mutation (48.9 years) (26). Indeed, in our cohort a single ATM germline mutation carrier was detected (1/86; 1.2%) that was over 40 years old. APC germline mutations account for the familial adenomatous polyposis (FAP) hereditary cancer syndrome that is characterized by the development of 100 to 1000 of colorectal adenomatous polyps. A germline *APC* mutation can be detected in approximately 80% of classic FAP cases, while 15% to 20% of FAP patients demonstrate *de novo* germline mutations. Our patient diagnosed with APC chr5: c.3920T>A (p.Ile1307Lys) germline mutation reported neither positive family history nor a co-existing colon or other GI malignancy. Indeed, one study that screened 1.462 sequential patients for multiple genes conferring inherited cancer predisposition reported incidental findings in 25 (1.7%) patients with little or no known personal or family history (27). APC germline mutations were detected in two patients with breast cancer and no family history or other malignancy resembling our results. However, the genetic polymorphism identified in our study c.3920T>A (p.Ile1307Lys) has been accused for increased risk for colorectal cancer in Ashkenazi Jewish patients (28).

Our study is characterized by certain strengths and limitations. The main strength of our study is the application of the powerful NGS analysis. NGS offers a revolutionary high-throughput method providing much shorter reads (~21 to ~400 base pairs) Instead of long reads generated from a PCR-amplified sample. This analysis enables the detection of not only high-penetrance genes with established clinical utility, but also genes with clinical significance that is less evident. The main limitation of our study is that it is confined to a single institution and thus the sample size is limited. The limited number of patients involved may account for the absence of statistical significance throughout the results reported. A multicenter study with a similar design could generate more robust scientific data. More studies with a larger sample size should be performed to confirm the above findings.

Overall, we here report the mutational profile of young females with breast cancer below 40 years of age compared to their older counterparts. No statistically significant differences were identified between the two groups, yet the sample size is relatively small to extract a safe conclusion. PIK3CA [6/20; 30%] and TP53 [6/20; 30%] were the most frequent pathogenic somatic mutations detected in this population, while CHEK2 [3/25; 12%] was the germline pathogenic mutation most frequently detected, especially the c.470T>C (rs17879961) gene polymorphism. Other common germline mutations were BRCA1/2 [3/25; 12%] and a single case of TP53 mutation [1/25; 4%]. Given the high incidence of pathogenic gene mutations, genetic testing of young patients with breast cancer could facilitate the therapeutic approach of the existing neoplasm, the prevention of secondary malignancies and the genetic counselling of their relatives.

## Data Availability Statement

The data can be found in medical records and patients' files and a single electronic database that was generated based on patients' files.

## Ethics Statement

The studies involving human participants were reviewed and approved by Institutional Review Board of the Alexandra General Hospital of Athens. The patients/participants provided their written informed consent to participate in this study.

## Author Contributions

AA: Data curation, Investigation, Original Draft Writing, Draft Editing-Review. SC: Data curation, Investigation. IK: Statistical analysis GB. Data Curation, Investigation. MK: Draft Editing-Review. ML: Data Curation, Investigation, Original Draft Writing. ML, M-AD, and FZ: Conceptualization, Project administration, Supervision, Draft Editing. All authors contributed to the article and approved the submitted version.

## Conflict of Interest

ML has received honoraria from Roche, Astra Zeneca, Astellas, MSD, Janssen, Bristol-Myers-Squibb and IPSEN. M-AD has received honoraria from participation in advisory boards from Amgen, Bristol-Myers-Squibb, Celgene, Janssen, Takeda. FZ has received honoraria for lectures and has served in an advisory role for Astra-Zeneca, Daiichi, Eli-Lilly, Merck, Novartis, Pfizer, and Roche.

The remaining authors declare that the research was conducted in the absence of any commercial or financial relationships that could be construed as a potential conflict of interest.

## Publisher’s Note

All claims expressed in this article are solely those of the authors and do not necessarily represent those of their affiliated organizations, or those of the publisher, the editors and the reviewers. Any product that may be evaluated in this article, or claim that may be made by its manufacturer, is not guaranteed or endorsed by the publisher.
