# Supplementary material for: The Mutational Landscape of Early-Onset Breast Cancer: A Next-Generation Sequencing Analysis
Source: Front Oncol. 2022 Jan 21;11:797505. doi: 10.3389/fonc.2021.797505 (PMC8813959; doi:10.3389/fonc.2021.797505)
Supplement: Supplementary file 2 [file Table_1.docx]

| AKT1 | BRCA1 | CDKN2A | ERBB4 | IDH2 | KRAS | MLH1 | NOTCH1 | PTEN | SMAD4 |
| --- | --- | --- | --- | --- | --- | --- | --- | --- | --- |
| ALK | BRCA2 | CHEK1 | ESR1 | JAK2 | MAP2K1 | MSH2 | NRAS | RAD50 | STAT3 |
| AR | CCND1 | CHEK2 | FGFR1 | JUN | MDM2 | MSH6 | PALB2 | RAD51 | STK11 |
| ATM | CCNE1 | CTNNB1 | FGFR2 | KDR | MDM4 | mTOR | PDGFRA | RB1 | TP53 |
| BCK2 | CDK4 | EGFR | FGFR3 | KIT | MEN1 | MYC | PIK3CA | RET |  |
| BRAF | CDK6 | ERBB2 | IDH1 | KMT2C | MET | NF1 | PIK3CB | ROS1 |  |

**Supplementary Table 1**. Gene panel tested in FFPE tissues

| APC | ATM | BAP1 | BLM | BMPR1A | BRCA1 | BRCA2 | BRIP1 | CDH1 | CDKN2A |
| --- | --- | --- | --- | --- | --- | --- | --- | --- | --- |
| CHEK2 | DICER1 | FANCM | FH | FLCN | MEN1 | MLH1 | MSH2 | MSH6 | MUTYH |
| NBN | NF1 | NF2 | PALB2 | PMS1 | PMS2 | PTEN | RAD51C | RAD51D | RB1 |
| RECQL4 | RET | SDHB | SDHC | SDHD | SLX4 | SMAD4 | STK11 | TP53 | TSC1 |
| TSC2 | VHL |  |  |  |  |  |  |  |  |

**Supplementary Table 2**. Gene panel analyzed in the germline NGS study
